# The efficacy of adjunctive alpha-blockers on ureteroscopy procedure for ureteral stones: a systematic review and meta-analysis

**DOI:** 10.12688/f1000research.52072.2

**Published:** 2022-04-12

**Authors:** Saras Serani Sesari, Widi Atmoko, Ponco Birowo, Nur Rasyid

**Affiliations:** 1 Department of Urology, Faculty of Medicine Universitas Indonesia, Cipto Mangunkusumo General Hospital, Jakarta, 10430, Indonesia

**Keywords:** adjunctive alpha-blocker, ureteral stone, ureteroscopy

## Abstract

**Background: **Urolithiasis cases are a common condition, and the number is still growing today. The prevalence of urinary tract stones globally currently ranges from 2-20% with a recurrence rate of around 50%. The present study aims to investigate the efficacy of adjunctive alpha-blockers in improving the success rate of ureteroscopy (URS) procedure for urolithiasis.

**Methods: **We reviewed articles obtained from MEDLINE, CENTRAL, CINAHL, and Elsevier from 14 August to 9 September 2020, comparing alpha-blockers as adjunctive therapy, versus either a placebo or no drug at all, in post-URS urolithiasis patients. There were no restrictions on the type of URS and alpha-blockers given to patients. The quality of studies included was assessed using Cochrane’s Risk of Bias Assessment for Randomized-Controlled Trials.

**Results: **Forest plot analysis emphasizes the statistically significant difference among the group, where the adjunctive alpha-blocker group had pooled relative risk (RR) of being stone-free, readmitted due to initial URS failure, having an overall complication, having haematuria, getting their ureteral mucous injured, and suffering a colic episode was 1.71 (95% CI, 1.11–1.24), 0.50 (95% CI, 0.25–1.01), 0.41 (95% CI, 0.27–0.61), 0.42 (95% CI, 0.22–0.79), 0.31 (95% CI, 0.13–0.73), and 0.21 (95% CI, 0.06–0.69), respectively.

**Conclusions: **Alpha blockers minimize the frequency and duration of ureteral contractions, allowing smooth stone expulsion. With this knowledge, it is expected to help clinicians decide the importance of adjunctive alpha-blocker administration.

## Introduction

In the last decade, urolithiasis has become a common condition, and the number continues to increase. The prevalence of urinary tract stones globally currently ranges from 2–20% with a recurrence rate of around 50%. The increase in urinary tract stones incidence was also followed by the rise in the frequency of urinary tract endoscopy, one of which was retrograde or antegrade ureterorenoscopy, which was indicated to treat ureteral stones and kidney stones ^1^. Compared to the extra-corporeal shock-wave lithotripsy (ESWL) procedure, (URS) is more preferred, as it has been proven to achieve higher success rates in a single operation ^2, 3^.

In recent literature, the adjunctive alpha-blocker is recommended to facilitate distal ureteric stone expulsion, decrease postoperative complications, improve stents tolerability, and reduce colic episodes to reduce the necessity for secondary procedure retreatment ^1^.

The present study aims to investigate the efficacy of adjunctive alpha-blockers for improving the success rate of the URS procedure for urolithiasis. By conducting this review and analysis, a definite conclusion regarding the effectiveness can be achieved. Thus, clinicians can decide the necessity of adjunctive alpha-blockers.

## Methods

### Description of condition

This review was done according to the Preferred Reporting Items for Systematic Reviews and Meta-analysis (PRISMA) statements ^4^. This study attempted to improve alpha-blocker therapy effectiveness in post-URS urolithiasis patients, with success rate parameters. Thus, this meta-analysis included studies which compared alpha-blockers as adjunctive therapy, versus either a placebo or no drug at all, in post-URS patients. No restrictions on the type of URS were performed in patients. There were also no restrictions on the kind of alpha-blocker given to patients. The success rate was then defined as the stone-free rate and overall postoperative complication rate.

### Database searching and literature screening

We performed an article search on four electronic databases (MEDLINE/Pubmed, CENTRAL/Cochrane, CINAHL/EBSCOHost, and EMBASE/Elsevier). The investigation was carried out from 14 August to 9 September 2020. PICOS were used to trace studies and identify the suitability of any we found ^5^. We used specific keywords, adjusted to each search engine specification, in the form of (postoperative OR adjunctive) AND (alpha-blocker OR tamsulosin OR alpha-adrenergic antagonists OR Alpha-adrenoreceptor antagonists OR doxazosin OR terazosin OR alfuzosin OR prazosin) AND (ureteroscopy OR URS OR ureterorenoscopy OR retrograde intrarenal surgery) AND ureteral stone. We also looked at a reference list of several reviews to expand the search coverage of the study.

### Study selection

Study selection was carried out independently and duplicated by each author, referring to inclusion and exclusion criteria. The inclusion criteria in this study included: 1) RCT or quasi-RCT studies that were compatible with PICOS; 2) English/Indonesian written articles; 3) Full-text articles available; 4) The output assessed were, at least, one of postoperative stone-free rate or overall complication rate; and 5) Published between 1 January 2000 and 31 December 2020.

There were no restrictions on the type of URS and alpha-blockers given to patients. The exclusion criteria for this study included review articles, case reports, case series, editorial letters, studies on animals, and/or studies in the process of peer review (has not been published yet).

The decision to study eligibility was determined by each author independently. Any disagreement was resolved by discussion.

### Data extraction and outcome of interest

Data extraction was carried out by each author independently and in duplication. We extracted the study's primary characteristics, including the first author, location, sample size, and publication year.

Following the dependent variables in this meta-analysis, we also extracted patient baseline data and postoperative data, including a stone-free rate and overall complication rate. We also noted the type of alpha-blocker and the duration of alpha-blocker administration.

This study explored the efficacy of adjunctive alpha-blockers in increasing URS's success rate in urolithiasis patients, divided into the stone-free rate and overall complication rate, in the form of relative risk (RR). We used a 2×2 contingency table to obtain each study's RRs and pooled the overall RRs using the Review Manager 5.3 application ^6^. Analysis using the DerSimonian and Laird random-effects model was performed when high heterogeneity was found.

### Study quality assessment

To see the precision and examine publication bias, this meta-analysis utilized funnel plots, which were also produced via Review Manager 5.3 ^6^.

## Results

### Literature findings

**Figure 1.**
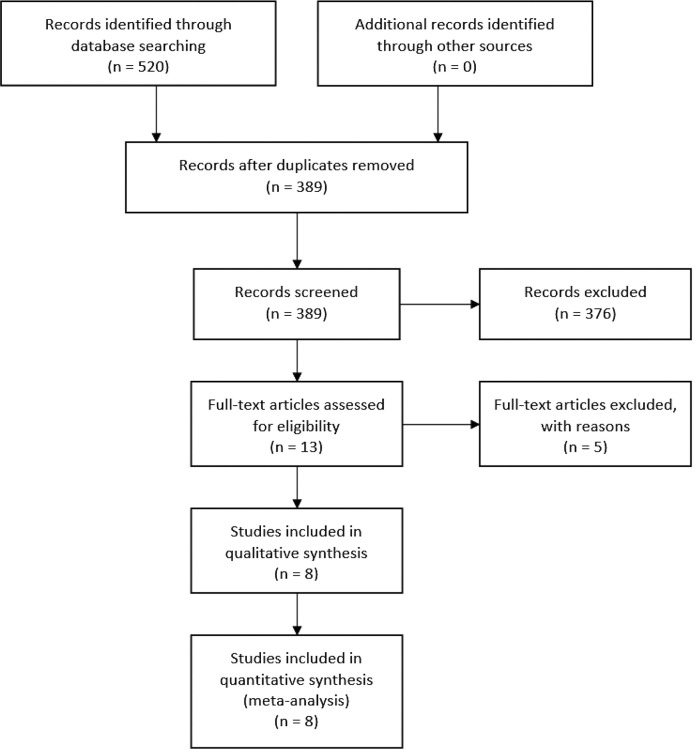
PRISMA flowchart detailing the article identification, screening, and inclusion process and results. Initial database searching yielded 520 items, 389 of which were left after duplicate screening. Abstract screening excluded 376 more items. Eight items survived full-text assessment and were included in both the qualitative and quantitative synthesis.

We searched five electronic databases, using specific keywords tailored to each database, to improve search sensitivity and specificity. The total records retrieved were 520 studies, and 131 studies were then excluded as there were duplications. From 389 studies screened, 376 studies were further excluded because of unrelated topics and objectives, resulting in 13 studies to be assessed for eligibility. Another five studies were later excluded due to unsuitable study design (n = 2) and review articles (n = 3). We obtained eight studies that were included in the qualitative and quantitative synthesis. The summary of study identification and selection according to the PRISMA Statement flow diagram are shown in Figure 1 and Table 1.

**Table 1.** Summary of article identification and exclusion from each database.

Database	Keywords	Hit	Selected	Comments
MEDLINE	((((ureteral stone) OR (urolithiasis) AND (clinicaltrial [Filter])) AND ((((ureteroscopy) OR (ureterorenoscopy)) OR (retrograde intrarenal surgery)) OR (URS) AND (clinicaltrial [Filter]))) AND ((((((((alpha-blocker) OR (tamsulosin)) OR (alpha-adrenergic antagonist)) OR (alpha-adrenoreceptor antagonist)) OR (terazosin)) OR (alfuzosin)) OR (doxazosin)) OR (prazosin) AND (clinicaltrial [Filter]))) AND (((postoperative) OR (adjunctive)) OR (additional) AND (clinicaltrial [Filter]))	12	4	8 not match PICOS
CENTRAL	"postoperative" OR "adjunctive" OR "additional" in Title Abstract Keyword AND "alpha-blocker" OR "tamsulosin" OR "alpha-adrenergic antagonist" OR "alpha-adrenoreceptor antagonist" OR "terazosin" OR "alfuzosin" OR "doxazosin" OR "prazosin" in Title Abstract Keyword AND "ureteroscopy" OR "ureterorenoscopy" OR "retrograde intrarenal surgery" OR "URS" in Title Abstract Keyword AND "ureteral stone" OR "urolithiasis" in All Text - (Word variations have been searched)	14	4	1 duplicated article 9 not match PICOS
EBSCO Host	(((postoperative) OR (adjunctive)) OR (additional)) AND ((((((((alpha- blocker) OR (tamsulosin)) OR (alpha-adrenergic antagonist)) OR (alpha-adrenoreceptor antagonist)) OR (terazosin)) OR (alfuzosin)) OR (doxazosin)) OR (prazosin)) AND ((((ureteroscopy) OR (ureterorenoscopy)) OR (retrograde intrarenal surgery)) OR (URS)) AND ((ureteral stone) OR (urolithiasis))	12	2	1 review article 1 pooled analysis article 8 not match PICOS
Scopus	ALL ("postoperative" OR "adjunctive" OR "additional") AND ALL ("alpha- blocker" OR "tamsulosin" OR "alpha-adrenergic antagonist" OR "alpha- adrenoreceptor antagonist" OR "terazosin" OR "alfuzosin" OR "doxazosin" OR "prazosin") AND ALL ("ureteroscopy" OR "ureterorenoscopy" OR "retrograde intrarenal surgery" OR "URS") AND ALL ("ureteral stone" OR "urolithiasis")	225	6	165 not match PICO 11 not written in English/ Bahasa 2 full-text not available 31 review articles
ProQuest	("postoperative" OR "adjunctive" OR "additional") AND ("alpha-blocker" OR "tamsulosin" OR "alpha-adrenergic antagonist" OR "alpha-adrenoreceptor antagonist" OR "terazosin" OR "alfuzosin" OR "doxazosin" OR "prazosin") AND ("ureteroscopy" OR "ureterorenoscopy" OR "retrograde intrarenal surgery" OR "URS") AND ("ureteral stone" OR "urolithiasis")	257	8	91 not match PICO 90 books/other sources 43 review articles 11 not written in English/ Bahasa 14 pilot studies

### Study characteristics

Three of the eight studies we included in this study were multicenter, prospective, randomized trials ^7– 9^. While the other five were a single-center, prospective, randomized trial ^1, 3, 10, 11^. However, the numbers of patients enrolled in the pilot studies did not differ significantly between studies. Overall, the total number of patients included in this meta-analysis was 913 patients.

Seven of the eight studies gave alpha-blockers before URS, three of those gave Tamsulosin 0.4 mg once daily for seven days before surgery ^1, 8, 10^. Two studies administered alpha-blockers only once, the day before surgery ^3, 9^. One study gave Silodosin 8 mg once daily for ten days before surgery ^10^, and one other study gave Tamsulosin 0.4 mg once daily for 14 days postoperatively ^11^. Bhattar *et al*. ^12^ also looked at Tamsulosin independently and in combinations. Data on the characteristics of the included studies are shown in Table 2.

**Table 2.** Characteristic of the studies included in this systematic review.

Author	Regimen	Timing	N	Age *	Duration	Design	Stone size * (mm)	Stone density * (HFU)
Ahmed *et al*. 2017. ^2^	Tamsulosin 0.4 mg qd	1 week preoperatively, discontinued after surgery	81/84	35.7±11.0/37.6±11.2	8 weeks	Prospective, randomized, multicentre study	13.37±2.73/12.73±2.39	815.35±341.6/778.36±269.2
Bayar *et al*. 2019. ^7^	Tamsulosin 0.4 mg qd	1 week preoperatively, discontinued after surgery	61/63	42.1±11.4/39±14.6	4 weeks	Prospective, randomized, multicentre study	NR	NR
Ketabchi *et al.* 2014. ^6^	Tamsulosin 0.4 mg qd	1 day preoperatively, discontinued after surgery	52/50	24±6.5/27±8.8	2 weeks	Prospective, randomized, single-centre study	6.6 ± 2.3/6.2 ± 3.2	NR
Aydin *et al*. 2018. ^8^	Silodosin 8 mg qd	1 day (A) and 3 days (B) preoperatively, discontinued after surgery	50/47/50	37 (22–65)/43 (22–78)/37.5 (20–69)	4 weeks	Prospective, randomized, multicentre study	NR	NR
Mohey *et al*. 2018. ^9^	Silodosin 8 mg qd	10 days preoperatively, discontinued after surgery	62/65	38.27±9.37/39.67±9.54	4 weeks	Prospective, randomized, single-centre study	12.6±1.25/12.9±1.29	907.66±208.52/898.97±212.04
Abdelazis *et al*. 2017. ^1^	Tamsulosin 0.4 mg qd	1 week preoperatively, discontinued after surgery	51/47	35/38	2 weeks	Prospective, randomized, single-centre study	6.6±2.3/6.2±3.2	NR
John *et al*. 2010. ^10^	Tamsulosin 0.4 mg qd	Initiated postoperatively for 2 weeks	40/38	36.9 (21-57)/42.2 (26-61)	4 weeks	Prospective, randomized, single-centre study	1.3 (1-2)/1.2 (1-1.8)	NR
Bhattar *et al*. 2018. ^11^	Tamsulosin 0.4 mg qd	10 days preoperatively, discontinued after surgery	36/36	35.42±11.4/37.1±10.9	4 weeks	Prospective, randomized, single-centre study	10.2±2.1/11.3±2.8	NR
Ahmed *et al*. 2017. ^2^	43.4±12.3/49.6±13.6	1.2±0.3/1.4±0.6	2.68 **†**/3.17 **††**	0.34	0.09	0.34	0.61	NR
Bayar *et al*. 2019. ^7^	33±9.4/30.9±6.6	NR	2.62	0.51	NR	NR	NR	NR
Ketabchi *et al*. 2014. ^6^	NR	NR	7.00	NR	NR	NR	0.51	0.16
Aydin *et al*. (A) 2018. ^8^	30 (10–45)/30 (15–75)	NR	1.00	0.31	NR	NR	NR	NR
Aydin *et al*. (B) 2018. ^18^	30 (10–50)/30 (15–75)	NR	5.15	0.07	NR	NR	NR	NR
Mohey *et al*. 2018. ^9^	41.61±4.67/46.85±4.6	NR	5.75	0.28	0.20	0.25	NR	NR
Abdelazis *et al*. 2015. ^1^	52.0±14.9/71.0±17.3	1.2±0.6/1.7±0.9	2.34	0.62	0.92	0.45	0.36	NR
John *et al*. 2010. ^10^	NR	NR	2.08	NR	NR	NR	NR	0.24
Bhattar *et al*. 2018. ^11^	34.41±6.76/45.20±6.963	2.12±0.33/2.56±0.93	2.67	NR	0.27	0.21	NR	NR

* Intervention/Control; ***mm***: millimeters ; ***qd***: drug was given once daily; ***HFU***: Hounsfield unit; ***NR***: not reported * I/C: Intervention/Control; ***comp***: complication; **†:** recorded at 4 weeks postoperatively; **††:** recorded at the end of study; ***NR***: not reported.

### Summary of bias risk

Of the eight studies included in this meta-analysis, five had a high risk of bias because assessor outcome blinding was not performed. One study clearly stated no blinding in patients who were given intervention or control ^11^. Selection bias in some studies was also considered high, because no allocation concealment was performed. In general, the quality of the studies included in this meta-analysis varied from low to high.

**Figure 2.**
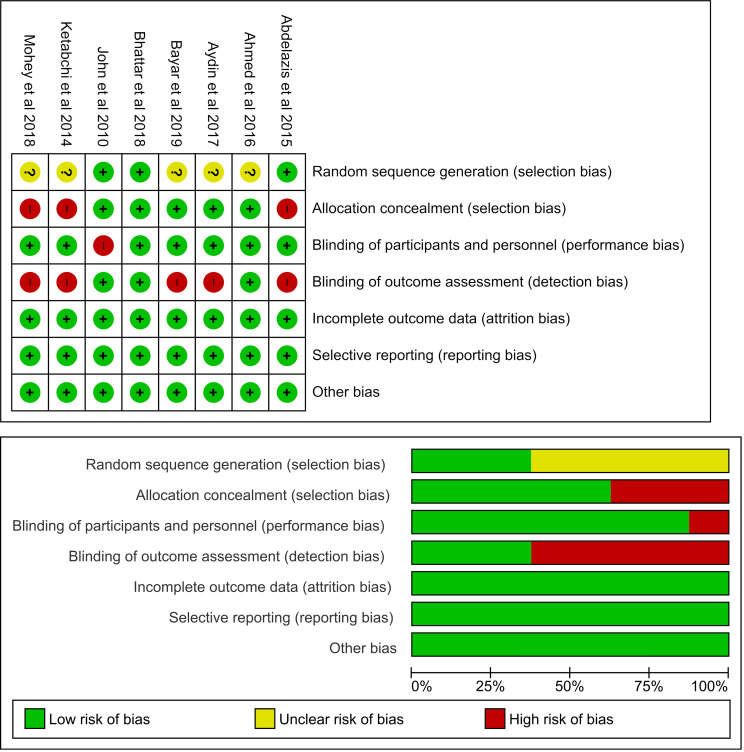
Risk of bias assessment summary using traffic light and summary plots. Selection bias from allocation concealment is present in three studies and not enough information was given to assess random sequence generation. Detection bias was detected in five studies. Only one study presented with performance bias. No attrition or reporting bias were detected.

The bias assessment was carried out independently by each author and in duplication, in which all authors assessed all articles. Disagreements between the authors were resolved by discussion or consensus. Figure 2 visualizes the summary of bias risk.

### Stone-free rate

The stone-free rate was found to be higher in patients given adjunctive alpha-blockers in most of the studies. A study by Aydin *et al.* 2017 ^9^, found no difference at all in the stone-free rates of patients with and without adjunctive alpha-blockers one day before URS surgery. However, this study also compared alpha-blocker administration three days before surgery with a placebo and found a significant difference between the placebo and alpha-blocker groups.

**Figure 3.**
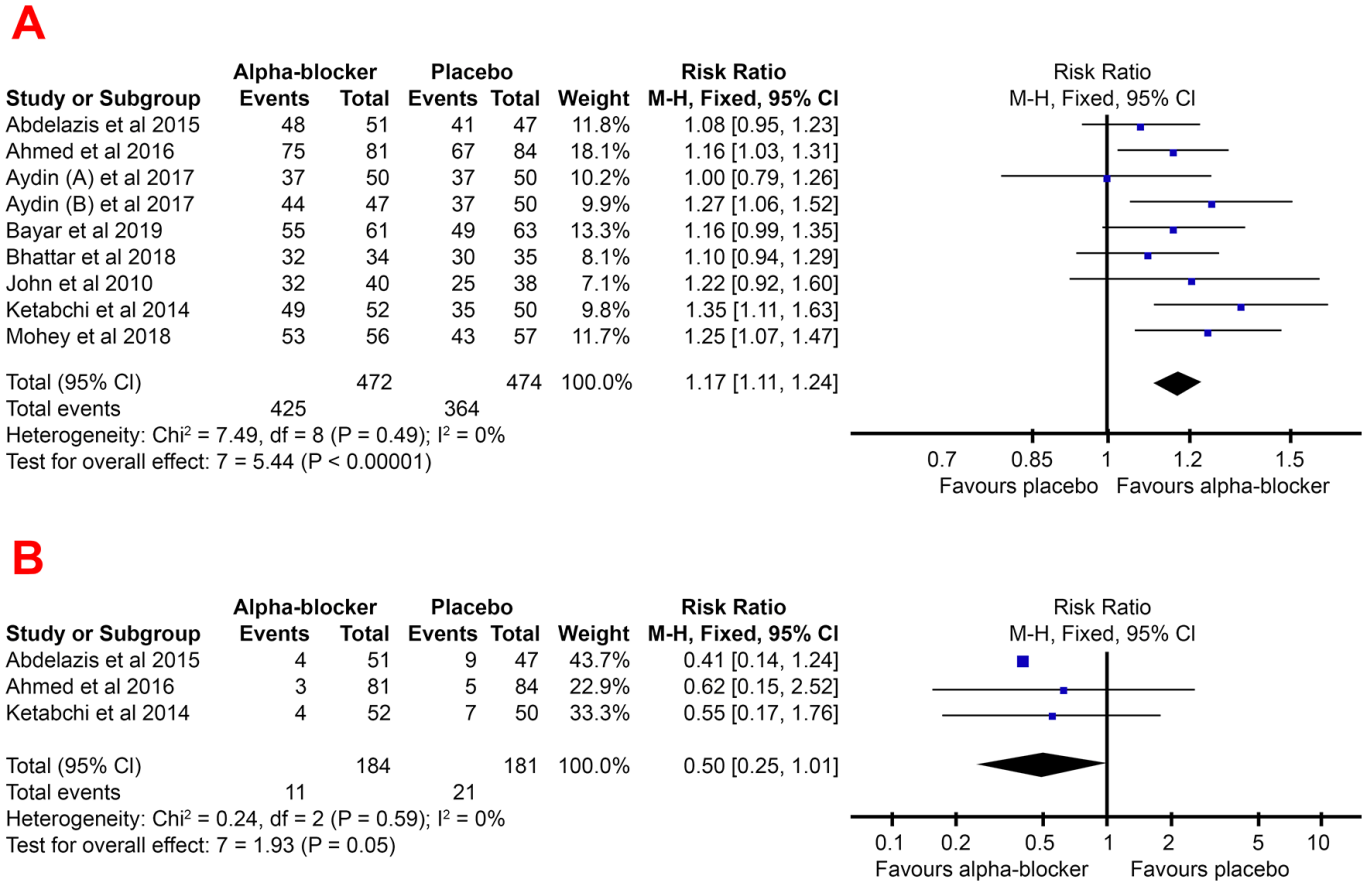
Forest plot of stone free rate ( **A**) and readmission ( **B**) pooled RR for the group treated with alpha-blocker compared to group treated with placebo -blocker compared to control group. The lower risk of readmissions due to initial URS failure also supported a higher stone-free rate in the alpha-blocker group. The risk of readmission to URS in the alpha-blocker group tends to be lower than the placebo group, with a pooled RR of 0.50 (95% CI, 0.25–1.01).

Based on the results of the meta-analysis in Figure 3, it could be seen that the pooled RR favors the experimental group (adjunctive alpha-blocker), with a value of 1.17 (95% CI, 1.11–1.24). This indicates that a significantly higher stone-free rate was found in the adjunctive alpha-blocker patient.

### Overall complication rate

In all studies, the complication rate was higher in the control group, either general complication or overall complication, hematuria, or mucosal injury. The alpha-blockers group had a significantly lower risk of complications than the placebo group, with a pooled RR of 0.41 (95% CI, 0.27–0.61). In this meta-analysis, the heterogeneity was recorded at only 0%; thus, we used a fixed-effect model to pool the effect estimate.

**Figure 4.**
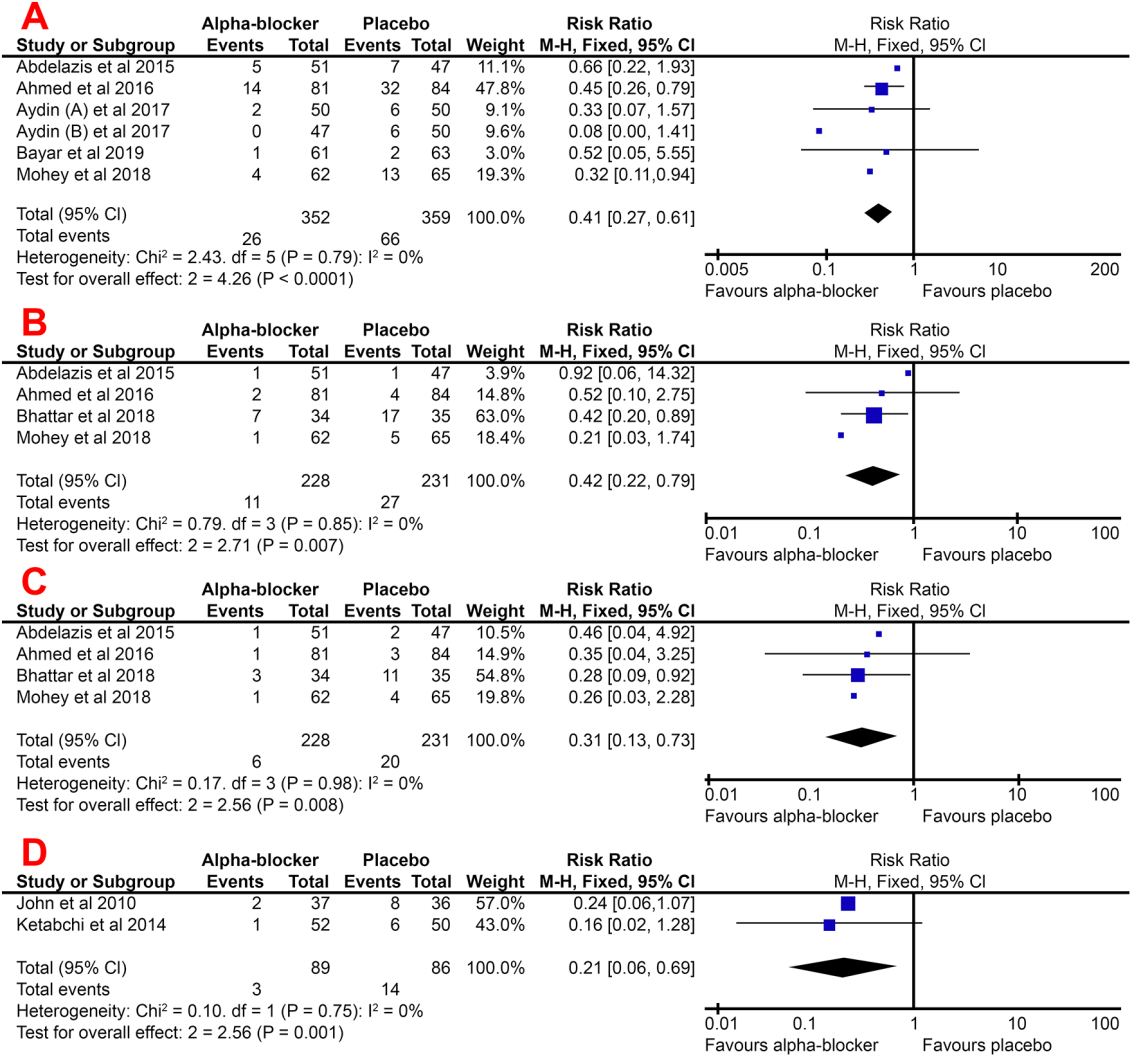
Forest plot of overall complication rate ( **A**), hematuria ( **B**), mucosal injury ( **C**), and colic episode ( **D**) pooled RR for the group treated with alpha-blocker compared to control group.

Several studies break down their patients' complications into several types of complications, such as hematuria, mucosal injury, and colic episode. For each of these complications, the alpha-blocker group was shown to have a lower risk of developing these complications. For all three types of complications, the alpha-blocker group had a significantly lower risk, with the pooled RR for hematuria, mucosal injury, and a colic episode of 0.42 (95% CI, 0.22–0.79), 0.31 (95% CI, 0.13–0.73), and 0.21 (95% CI, 0.06–0.69), respectively. These are summarized in Figure 4.

## Discussion

According to this systematic review and meta-analysis, postoperative alpha-blocker is associated with URS procedure's success rate for urolithiasis ^2, 13^. From the forest plot, it was discovered that patients having postoperative alpha-blockers are more likely to be stone-free with the RR=1.17 (95% CI, 1.11–1.24). This means that patients consuming postoperative alpha-blockers are 1.17 times more likely to be stone-free. The risk ratio ranges from 1.11–1.24, which are both greater than 1. Thus, it can be concluded that postoperative alpha-blocker is effective in increasing the stone-free rate after ureteroscopy. The study with the highest weight is by Ahmed *et al*. 2017 ^2^, with a weight of 72.2%, followed by a study by Bayar *et a*l. 2019 ^8^, with a weight of 27.8%. This result is similar to a study done by Alsaikhan *et al*. in 2020 ^14^, albeit using different parameters. They did a systematic review and meta-analysis about preoperative alpha-blockers usage for ureteroscopy for ureteric stones with parameters including risk reduction in need for intraoperative ureteral dilatation, stone-free status at four weeks post-operatively, and at final follow-up, the likeliness of urologists to reach the stone with the ureteroscope, operative time, and length of hospital stay. They study the preoperative use of alpha-blockers, meanwhile this study analyzed the usage of alpha-blockers as an adjunctive both preoperatively and postoperatively. The study result showed that at four weeks post-operatively and at final follow-up, patients have increased stone-free status with RR 1.17 (95% CI: 1.08 to 1.26), p < 0.0001 and 1.18 (95% CI: 1.11 to 1.24), p < 0.00001 respectively. It is important to note that some studies that were assessed by this study and Alsaikhan *et al.* 2020 are different ^14^.

Tamsulosin, an alpha-1A blocker, has been shown to improve the distal ureteral stone expulsion rate and minimize the probability of colic episodes in patients during watchful waiting ^15, 16^. It relaxes the muscle of the distal ureteral wall, aiding gravel clearance after URS or ESWL procedure. Furthermore, a relaxed ureter allows the instrument forwarding to become easier. In patients with Tamsulosin, the ureteral orifices were dilated, easily identified, and provided a more accessible entrance for the ureteroscope ^8, 17^. Tamsulosin also lessens the amplitude of ureteral contractions and shortens the duration between contractions ^15, 18^.

Postoperative complications outcome is also affected by the administration of postoperative alpha-blockers ^19, 20^. It was found that endoscopic treatments without the administration of adjunctive alpha-blockers are associated with a higher probability of complications ^2, 21^. This was shown by the forest plot, where the adjunctive alpha-blocker gives a protective effect from postoperative complications with RR = 0.41 (95% CI, 0.27–0.61). Therefore, postoperative alpha-blocker administration reduces the odds of postoperative complications. For this outcome, a study by Mohey *et al*. 2018 ^10^, weighs 19.3%, while a study by Ahmed *et al*. 2017 ^2^ weighs 47.8%. Extra benefits, such as shorter hospital stay, lesser hospital bills, milder postoperative complications, and better symptomatic improvement were obtained by simultaneous administration of Tamsulosin ^18, 20^.

Postoperative alpha-blocker improves the incidence of colic episodes. This was shown by RR=0.21 (95% CI, 0.06–0.69). Numerous reports have demonstrated alpha-adrenoreceptors on the ureteral wall, with the distal ureter's highest density ^1, 2, 15^. Variable distribution of alpha receptor subtypes were found in the proximal, middle, and distal ureter. Alpha-blockers are now commonly prescribed for ureteral colic in various hospitals ^15, 22^.

**Figure 5.**
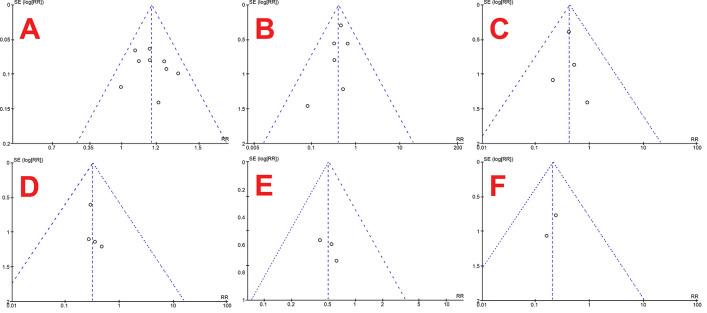
Funnel plots of stone-free rate ( **A**), overall complication rate ( **B**), hematuria ( **C**), mucosal injury ( **D**), readmission rate ( **E**) and colic episodes ( **F**). Aside from the strengths, this systematic review and meta-analysis has a limitation. This study's limitation is that there were only eight studies eligible for review, and there were only two studies for each outcome, which makes its representability somewhat questionable.

This systematic review and meta-analysis have several strengths. Firstly, this systematic review and meta-analysis included a relatively broad scope of population. This review then assesses the primary outcome and considers other additional outcomes, which is also essential in clinical practices, albeit not widely studied. Low risk of bias in included studies, utilization of guidelines, no heterogeneity between studies, symmetrical funnel plots as shown in Figure 5, and high specificity also strengthen this study.

## Conclusions

In conclusion, our study shows that the administration of adjunctive alpha-blockers improves the URS procedure's success rate for ureteral calculi in terms of increasing stone-free rate, reducing postoperative complications, and minimizing colic episodes. This is because the alpha-blocker relaxes and reduces the ureteral wall's contractions, allowing easier stone clearance. Alpha-blockers have been shown to enhance the rate of distal ureteral stone ejection. The use of -blockers in the conservative therapy of distal ureteral stones is advised in the appropriate clinical context.

## Data availability

Open Science Framework: PRISMA checklist and flow chart for 'The efficacy of adjunctive alpha-blocker on ureteroscopy procedure for ureteral stones: a systematic review/meta-analysis'

DOI 10.17605/OSF.IO/RM4AG ^4^

Data are available under the terms of the Creative Commons Zero "No rights reserved" data waiver (CC0 1.0 Public domain dedication).

## References

[1] Abdelaziz AS Kidder AM : Tamsulosin therapy improved the outcome of ureterorenoscopy for lower ureteral stones: A prospective, randomised, controlled, clinical trial. *Afr J Urol.* 2017;23(2):148–53. 10.1016/j.afju.2015.12.003

[2] Ahmed AF Maarouf A Shalaby E : Semi-rigid ureteroscopy for proximal ureteral stones: Does adjunctive tamsulosin therapy increase the chance of success? *Urol Int.* 2017;98(4):411–7. 10.1159/000452926 27871076

[3] Rashahmadi N Sofimajidpour H Saedi A : The effect of Tamsulosin on the quality and clinical trial. *Discovery.* 2018;22(91).

[4] Serani S : PRISMA checklist and flow chart for ‘The efficacy of adjunctive alpha-blocker on ureteroscopy procedure for ureteral stones: a systematic review/meta-analysis’. *OSF.* 2021. 10.17605/OSF.IO/RM4AG PMC9021686 35464176

[5] Eriksen MB Frandsen TF : The impact of patient, intervention, comparison, outcome (PICO) as a search strategy tool on literature search quality: a systematic review. *J Med Libr Assoc.* 2018;106(4):420–431. 10.5195/jmla.2018.345 30271283 PMC6148624

[6] Review Manager (RevMan) [Computer program]: *Version 5*. Copenhagen: The Nordic Cochrane Centre, The Cochrane Collaboration.2014;3. Reference Source

[7] Ketabchi AA Mehrabi S : The effect of tamsulosin, an alpha-1 receptor antagonist as a medical expelling agent in success rate of ureteroscopic lithotripsy. *Nephrourol Mon.* 2013;6(1):e12836. 10.5812/numonthly.12836 24719805 PMC3968957

[8] Bayar G Kilinc MF Yavuz A : Adjunction of tamsulosin or mirabegron before semi-rigid ureterolithotripsy improves outcomes: prospective, randomized single-blind study. *Int Urol Nephrol.* 2019;51(6):931–6. 10.1007/s11255-019-02142-0 30989563

[9] Aydın M Kılınç MF Yavuz A : Do alpha-1 antagonist medications affect the success of semi-rigid ureteroscopy? A prospective, randomised, single-blind, multicentric study. *Urolithiasis.* 2018;46(6):567–72. 10.1007/s00240-017-1026-6 29151116

[10] Mohey A Gharib TM Alazaby H : Efficacy of silodosin on the outcome of semi-rigid ureteroscopy for the management of large distal ureteric stones: blinded randomised trial. *Arab J Urol.* 2018;16(4):422–8. 10.1016/j.aju.2018.07.002 30534442 PMC6277265

[11] John TT Razdan S : Adjunctive tamsulosin improves stone free rate after ureteroscopic lithotripsy of large renal and ureteric calculi: a prospective randomized study. *Urology.* 2010;75(5):1040–2. 10.1016/j.urology.2009.07.1257 19819530

[12] Bhattar R Tomar V Yadav SS : Comparison of safety and efficacy of tamsulosin, tadalafil, combinations and deflazacort in lower ureteric orifice negotiation by large size ureteroscope (8/9.8Fr) prior to intracorporeal lithotripsy. *Afr J Urol.* 2018;24(2):139–45. 10.1016/j.afju.2018.01.001

[13] Ramesh A Karthick P Kumar R : Medical expulsion therapy for ureteric calculus - possible! *F Int Surg J.* 2016;3(1):113–8. 10.18203/2349-2902.isj20160210

[14] Alsaikhan B Koziarz A Lee JY : Preoperative alpha-blockers for ureteroscopy for ureteral stones: a systematic review and meta-analysis of randomized controlled trials. *J Endourol.* 2020;34(1):33–41. 10.1089/end.2019.0520 31507224

[15] John TT Razdan S : Adjunctive tamsulosin improves stone free rate after ureteroscopic lithotripsy of large renal and ureteric calculi: a prospective randomized study. *Urology.* 2010;75(5):1040–2. 10.1016/j.urology.2009.07.1257 19819530

[16] Li S Penniston K : Combination versus monotherapy with alpha-blockers and anticholinergics for the relief of urinary stent symptoms - a randomized control trial.2017;1–15. Reference Source

[17] Lim KT Kim YT Lee TY : Effects of tamsulosin, solifenacin, and combination therapy for the treatment of ureteral stent related discomforts. *Korean J Urol.* 2011;52(7):485–8. 10.4111/kju.2011.52.7.485 21860770 PMC3151637

[18] Aydın M Kılınç MF Yavuz A : Do alpha-1 antagonist medications affect the success of semi-rigid ureteroscopy? A prospective, randomised, single-blind, multicentric study. *Urolithiasis.* 2018;46(6):567–72. 10.1007/s00240-017-1026-6 29151116

[19] Ata ur Rehman DrR Muzammil Tahir DrM Seerwan DrM : Effect of Tamsulosin on Stentrelated Symptoms; a Prospective Study. *Profession Med J.* 2016;23(01):114–118.

[20] Zhu J Liang Y Chen W : Effect of alpha1-blockers on stentless ureteroscopic lithotripsy. *Int Braz J Urol.* 2016;42(1):101–6. 10.1590/S1677-5538.IBJU.2014.0478 27136474 PMC4811233

[21] Navanimitkul N Lojanapiwat B : Efficacy of tamsulosin 0.4 mg/day in relieving double-J stent-related symptoms: A randomized controlled study. *J Int Med Res.* 2010;38(4):1436–41. 10.1177/147323001003800425 20926016

[22] Ferrandino MN Monga M Preminger GM : Adjuvant therapy after surgical stone management. *Adv Chronic Kidney Dis.* 2009;16(1):52–59. 10.1053/j.ackd.2008.10.007 19095206

