# Supplementary material for: The efficacy of adjunctive alpha-blockers on ureteroscopy procedure for ureteral stones: a systematic review and meta-analysis
Source: F1000Res. 2022 Apr 12;10:427. Originally published 2021 May 28. [Version 2] doi: 10.12688/f1000research.52072.2 (PMC9021686; doi:10.12688/f1000research.52072.2)
Supplement: Peer review history

# Peer review: Reviewer response for version 2

**DOI:** 10.5256/f1000research.122564.r134710

**Published:** 2022-04-20

**Authors:** Ferry Safriadi

**Affiliations:** 1 Department of Urology, Hasan Sadikin Academic Medical Center, Faculty of Medicine, Padjadjaran University, Bandung, Indonesia

After the authors revised this article, I agree to index the article.

Are the rationale for, and objectives of, the Systematic Review clearly stated?

Yes

Is the statistical analysis and its interpretation appropriate?

I cannot comment. A qualified statistician is required.

Are sufficient details of the methods and analysis provided to allow replication by others?

Partly

Are the conclusions drawn adequately supported by the results presented in the review?

Yes

Reviewer Expertise:

Endourology and oncology urology

I confirm that I have read this submission and believe that I have an appropriate level of expertise to confirm that it is of an acceptable scientific standard.

# Peer review: Reviewer response for version 1

**DOI:** 10.5256/f1000research.55298.r125918

**Published:** 2022-03-14

**Authors:** Evangelos Liatsikos

**Affiliations:** 1 Department of Urology, University of Patras Medical School, Rio, Greece

I have several comments for the authors.

1. The authors should clearly indicate the aim of the study in the Introduction.
2. The authors mention in the study selection that studies were selected if full-text articles were available. So they might have missed many important articles which full text was not available.

Are the rationale for, and objectives of, the Systematic Review clearly stated?

Yes

Is the statistical analysis and its interpretation appropriate?

Yes

Are sufficient details of the methods and analysis provided to allow replication by others?

Yes

Are the conclusions drawn adequately supported by the results presented in the review?

Yes

Reviewer Expertise:

Urology

I confirm that I have read this submission and believe that I have an appropriate level of expertise to confirm that it is of an acceptable scientific standard.

# Peer review: Reviewer response for version 1

**DOI:** 10.5256/f1000research.55298.r88750

**Published:** 2021-07-28

**Authors:** Ferry Safriadi

**Affiliations:** 1 Department of Urology, Hasan Sadikin Academic Medical Center, Faculty of Medicine, Padjadjaran University, Bandung, Indonesia

This study is interesting. Some limitations I see in this study are:

1. Only 2 studies with multicenter and each outcome parameter.
2. In this review, it is not mentioned about stone location in the ureter, which is in a proximal, mid ureter, or distal ureter. As we know, an alpha-blocker only works in the distal part of ureter. 

Based on that, the author should say this study is only good for distal ureter stones.

Are the rationale for, and objectives of, the Systematic Review clearly stated?

Yes

Is the statistical analysis and its interpretation appropriate?

I cannot comment. A qualified statistician is required.

Are sufficient details of the methods and analysis provided to allow replication by others?

Partly

Are the conclusions drawn adequately supported by the results presented in the review?

Yes

Reviewer Expertise:

Endourology and oncology urology

I confirm that I have read this submission and believe that I have an appropriate level of expertise to confirm that it is of an acceptable scientific standard, however I have significant reservations, as outlined above.

# Response

**Published:** 2022-03-26

**Authors:** Saras Serani Sesari

**Affiliations:** Department of Urology, Universitas Indonesia, Indonesia

I already added the clarification of which alpha-blockers exert their effects on the distal region of the ureter muscle. Because kidney and urinary stones can occur in a variety of locations, it was vital to state this explicitly. The author hopes that by identifying the place in which alpha-blockers are effective, which was distal ureter, this study will be easier to implement in the future.
